# The study of bone mineral density measured by quantitative computed tomography in middle-aged and elderly men with abnormal glucose metabolism

**DOI:** 10.1186/s12902-022-01076-6

**Published:** 2022-07-07

**Authors:** Pei Zhang, Xiaofeng Huang, Yanping Gong, Yanhui Lu, Minyan Liu, Xiaoling Cheng, Nan Li, Chunlin Li

**Affiliations:** 1 School of Medicine, Nankai University, Tianjin, 300071 China; 2 Department of Endocrinology, the Second Medical Center, the People’s Liberation Army General Hospital, National Clinical Research Center for Geriatric Disease, Fuxing Road 28, Beijing, 100853 China

**Keywords:** QCT, Bone mineral density, Aging men, Glucose metabolism

## Abstract

**Background:**

To investigate the value of quantitative computed tomography (QCT) measurement of bone mineral density (BMD) in middle-aged and elderly men with abnormal blood glucose.

**Methods:**

Total 138 men aged above 50 years old with routine physical examination were involved in the study. All participants were evaluated with serological index, BMD by QCT and dual energy X-ray absorptiometry (DXA). Statistical analysis was conducted by χ^2^ test and regression model.

**Results:**

All individuals were divided into normal blood glucose (NBG) group and abnormal blood glucose (ABG) group. Compared with NBG group, BMD detected by QCT was obviously lower in ABG group (*P* < 0.05). More cases with low bone mass or osteoporosis were detected by QCT measurement than DXA (χ^2^ = 56.618, *P* = 0.000), which were consistent in both NBG and ABG groups (χ^2^ was 33.564 and 23.250, *P* = 0.000). No significant difference of detection rates was found in both subgroups neither by QCT nor DXA measurement (χ^2^ = 4.204, *P* = 0.122). Regression analysis revealed that ABG was negatively associated with BMD assessed by QCT (β = -0.217, *P* < 0.05), which did not show statistical difference after adjusting for age.

**Conclusion:**

In middle-aged and elderly male patients with NBG or ABG, QCT manifests higher detection rates of low bone mass and osteoporosis than DXA. ABG is negatively correlated with BMD measured by QCT, which is affected by age.

## Introduction

Type 2 diabetes mellitus (T2DM) and Osteoporosis (OP) are common chronic diseases worldwide in middle-aged and elderly individuals. Osteoporosis is a systemic bone disease in which reduced bone mass and degradation of bone microstructure increase bone fragility and fracture risk, leading to the substantial economic burden.^1^ Meanwhile, as population ages, the burden of diabetes and fractures caused by osteoporosis is also aggravating. It is important to identify patients at high risk of osteoporosis and further administrate treatment early to prevent fractures. Although bone mineral density (BMD) in patients with diabetes might be increased, decreased or normal, the fracture risk is significantly increased.^2^ Therefore, the diagnosis of osteoporosis in patients with abnormal glucose metabolism is particularly significant.

Detection of BMD is incredibly central component to confirm the diagnosis of osteoporosis, of which DXA and QCT are of great importance. BMD measured by dual energy X-ray absorptiometry (DXA) is the current gold standard for diagnosis of osteoporosis nowadays, which is simple, fast, low-radiation and conveniently operated. Quantitative computed tomography (QCT) also plays a significant role in the prediction, diagnosis, treatment and prevention of osteoporosis and fractures.^3,4^ Volumetric bone density measured by QCT is more sensitive to changes in BMD than by DXA, which measures area bone density.

At present, there are numerous studies associated with osteoporosis in women, but evidence of BMD associated with QCT measurement in men is extremely scare, especially in patients with abnormal blood glucose (ABG). Therefore, aims of the study were to compare the detection rates of osteoporosis in middle-aged and elderly men with ABG and normal blood glucose (NBG) by DXA and QCT and to evaluate the significance of QCT application in middle-aged and elderly men with ABG.

## Material and methods

### Participants

All enrolled participants were aged over 50 and regularly attended to routine physical examinations at the People’s Liberation Army General Hospital from August 2017 to August 2018. The exclusion criteria were: (1) malignant tumor, severe hepatic insufficiency, severe renal insufficiency, moderate-severe anemia, acute cardiovascular and cerebrovascular diseases, and patients with metabolic bone diseases such as hyperparathyroidism, (2) medication therapy such as testosterone, glucocorticoids and vitamin D within 3 months, or anti-osteoporosis drugs such as bisphosphonate, parathyroid hormone, calcitonin and vitamin K within 12 months, (3) those without biochemical examination, DXA examination or QCT examination, or the interval of DXA and QCT examination is more than 1 month.

Our study was approved by the Medical Ethics Committee of the Chinese People's Liberation Army General Hospital (07 October 2014) and consistent with the Declaration of Helsinki. Written informed consent was acquired from all participated patients.

### Grouping and Clinical data

The demographic data, medical history and medication were obtained from electronic medical records. Laboratory indicators were detected, including fasting plasma glucose (FPG), alanine transaminase (ALT), creatinine (Cr), serum calcium (sCa), serum inorganic phosphorus (sPi), glycated hemoglobin (HbA1c), parathyroid hormone (PTH), 25-hydroxy vitamin D (25OHD), cross linked C telopeptide of type 1 collagen (CTX), amino-terminal propeptide of type 1 collagen (P1NP) and blood routine.

All participants were divided into NBG group and ABG group according to the medical history and diagnostic criteria of the World Health Organization (WHO).^5^

NBG refers to FPG < 6.1 mmol/L and 2-h postprandial plasma glucose (2hPG) < 7.8 mmol/L. ABG includes impaired fasting blood glucose (6.1 mmol/L ≤ FPG < 7.0 mmol/L and 2hPG < 7.8 mmol/L), impaired glucose tolerance (FPG < 7.0 mmol/L and 7.8 mmol/L ≤ 2hPG < 11.1 mmol/L) and diabetes (FPG ≥ 7.0 mmol/L and 2hPG ≥ 11.1 mmol/L).

### BMD determination and diagnostic criteria

Prodigy Advance DXA instrument from GE-LUNAR corporation was used to detect the BMD of lumbar (L2-4) vertebrae and femur neck (FN), and the unit of which is g/cm^2^. Then, the T scores were calculated based on the measured BMD. The diagnosis of low bone mass and osteoporosis was referred to the criteria of WHO^6^: (1) normal bone mass (NBM) is identified by the value for BMD or bone mineral content (BMC) that is not more than 1SD below the young adult mean value (T value > -1), (2) low bone mass (LBM) is identified by the value for BMD or BMC that lies between 1 and 2.5SD below the young adult mean value (-2.5 < T value ≤ -1), (3) osteoporosis is identified by the value for BMD or BMC that is more than 2.5SD below the young adult mean value (T value ≤ -2.5).

The 64-slice multi-slice spiral CT machine from GE corporation and the 5-sample solid phantom from Mindways were used to measure BMD. The original images were analyzed by Mindways QCT Pro software, in which L1-3 vertebral BMD, not including vertebrae with fractures, compression or deformation, were measured. Diagnosis of osteoporosis is based on the average value of measured vertebral BMD by QCT, diagnostic criteria of which is followed by the International Society for Clinical Densitometry (ISCD): BMD > 120 mg/cm^3^ means NBM, 80–120 mg/cm3 means LBM, and < 80 mg/cm3 means osteoporosis.^7^

### Statistical analysis

All statistical analyses were performed by SPSS 25.0 software. Normally distributed data were presented as x ± s. The comparison between two groups was construed by t test. Linear regression was used to analyze the factors associated with BMD. Paired χ^2^ test was used to compare the detection rates of osteoporosis by DXA and QCT. The detection rate of osteoporosis between NBG group and ABG group was analyzed by using χ^2^ test. *P* < 0.05 indicates that the difference is statistically significant.

## Results

### Demographic data in middle-aged and elderly men with NBG or ABG

A total of 138 middle-aged and elderly men were included, and all aged 50.9–95.0 years old. The average age of total individuals was (70.67 ± 12.32) years old. All 138 participants were divided into two groups based on the medical history and/or the clinical diagnosis, of which 97 were in the NBG group (70.3%) and 41 were in the ABG group (29.7%). All general data were compared between two groups and results were shown in Table 1. The average age in NBG group was significantly lower than the ABG group (68.80 ± 12.40 vs. 75.09 ± 11.08) (*P* < 0.05). The FPG in NBG and ABG group were (5.79 ± 0.89) mmol/L and (6.51 ± 1.08) mmol/L, and HbA1c were (5.85 ± 0.45) % and (6.50 ± 0.91) %, respectively. Consistent with the grouping, the FPG and HbA1c in NBG group were lower than the ABG group (*P* < 0.05). No significant difference was found in BMI, ALT, Cr, sCa, sPi, P1NP, CTX, 25OHD and PTH between two groups (*P* > 0.05) (Table 1).

**Table 1:** Demographic data and laboratory indicators in NBG and ABG group (x ± s)

	Total	NBG	ABG	*P* value
	138 (100%)	97 (70.3%)	41 (29.7%)	
Age	70.67 ± 12.32	68.80 ± 12.40	75.09 ± 11.08	0.006*
BMI (kg/m^2^)	24.55 ± 3.21	24.41 ± 3.19	24.87 ± 3.26	0.444
ALT (U/L)	19.12 ± 8.73	18.57 ± 8.05	20.44 ± 10.16	0.251
Cr (μmol/L)	79.85 ± 13.98	78.70 ± 13.94	82.56 ± 13.89	0.139
sCa (mmol/L)	2.36 ± 0.08	2.36 ± 0.08	2.36 ± 0.08	0.962
sPi (mmol/L)	1.05 ± 0.12	1.05 ± 0.10	1.05 ± 0.15	0.837
FPG (mmol/L)	6 ± 1	5.79 ± 0.89	6.51 ± 1.08	0.000*
HbA1c (%)	6.03 ± 0.62	5.85 ± 0.45	6.50 ± 0.91	0.000*
P1NP (ng/mL)	33.07 ± 11.43	30.18 ± 10.34	35.41 ± 12.06	0.227
CTX (ng/mL)	0.31 ± 0.2	0.26 ± 0.12	0.30 ± 0.25	0.227
25OHD (ng/mL)	22.83 ± 10.24	20.79 ± 6.89	22.48 ± 12.29	0.343
PTH (pg/mL)	42.15 ± 13.63	42.13 ± 14.61	39.86 ± 12.80	0.302

Notes: All *P < 0.05

*Abbreviations*: *NBG* Normal blood glucose group; *ABG* Abnormal blood glucose group; *BMI* Body mass index; *ALT* Glutamic-pyruvic transaminase; *Cr* Creatinine; *sCa* Serum Calcium; *sPi* Serum inorganic Phosphorus; *FPG* Fasting blood glucose; *HbA1c* Glycated hemoglobin; *P1NP* Amino-terminal propeptide of type 1 collagen; *CTX* Cross linked C telopeptide of type 1 collagen; *25OHD* 25-hydroxy vitamin D; *PTH* Parathyroid hormone

### Comparison of BMD and detection rates of OP measured by DXA and QCT in middle-aged and elderly men with NBG or ABG

BMD measured by DXA and QCT were compared between middle-aged and elderly men with NBG or ABG, results of which were presented in Table 2. The BMD of L2-4 vertebrae (1.24 ± 0.21 vs. 1.20 ± 0.21 g/m^2^) and FN (0.92 ± 0.12 vs. 0.91 ± 0.14 g/m^2^) measured by DXA, were slightly higher in ABG group than NBG group, but the difference did not reach statistical difference (*P* > 0.05). Then, the BMD measured by QCT in ABG group was significantly lower than in NBG group (96.73 ± 29.72 vs. 111.21 ± 33.76 g/m^3^) (*P* < 0.05).

**Table 2:** Comparison of BMD between NBG and ABG group (x ± s)

	NBG	ABG	*P* value
DXA BMD in lumbar vertebrae	1.20 ± 0.21	1.24 ± 0.21	0.341
DXA BMD in FN	0.91 ± 0.14	0.92 ± 0.12	0.809
QCT BMD	112.25 ± 33.93	96.57 ± 29.85	0.018*

*Notes*: All *P < 0.05

*Abbreviations*: *NBG* Normal blood glucose group; *ABG* Abnormal blood glucose group; *DXA* Dual energy X-ray absorptiometry; *QCT* Quantitative computed tomography; *BMD* Bone mineral density; *L1-4* Lumbar 1–4; *FN* Neck of femur

The detection rates of LBM and OP measured by DXA and QCT were analyzed. Firstly, for the detection rates by QCT measurement, there were 69 (50.0%) individuals with LBM and 27 (19.6%) with OP, with a total of 96 cases (69.6%) in total population. Meanwhile, in the NBG group, 49 (50.5%) cases were detected by LBM and 15 (15.5%) cases were detected by OP, a total of 64 (66%) cases. In the ABG group, 20 (48.8%) cases were detected by LBM and 12 (29.3%) cases were detected by OP, a total of 32 (78.1%) cases. There was no significant difference in detection rates of LBM and OP between NBG and ABG groups (χ^2^ = 4.204, *P* = 0.122). Moreover, we also analyzed the detection rates of DXA for LBM and OP, mainly including lumbar vertebrae and FN. In total 138 patients, 43 cases with LBM (31.2%) and 4 cases with OP (2.9%) were diagnosed by DXA, with a total of 47 cases (34.1%). Furthermore, in the NBG group, 31 (32.0%) cases were diagnosed by LBM and 3 (3.1%) cases were diagnosed by OP, a total of 34 (35.1%) cases. In the ABG group, 12 (29.3%) cases were diagnosed by LBM and 1 (2.4%) case were diagnosed by OP, a total of 13 (31.7%) cases. No statistical difference of detection rates was found between two groups based on DXA (χ^2^ = 0.158, *P* = 0.924). Then the detection rates of different measurements were further compared between ABG and NBG groups. Compared with DXA method, QCT extraordinarily elevated the detection rates of LBM and OP in total 138 population (χ^2^ = 56.618, *P* = 0.000). Higher LBM and OP detection rates by QCT than DXA measurement were also shown in NBG group (χ^2^ = 33.564, *P* = 0.000) and ABG group (χ^2^ = 23.250, *P* = 0.000) (Table 3).

**Table 3:** LBM and OP detection rates by QCT and DXA in different glucose metabolic status [cases (%)]

		NBG (97, 70.3%)	ABG (41, 29.7%)	Total (138, 100%)
QCT BMD	NBM	33(34.0%)	9(21.9%)	42(30.4%)
	LBM	49(50.5%)	20(48.8%)	69(50.0%)
	OP	15(15.5%)	12(29.3%)	27(19.6%)
DXA BMD	NBM	63(64.9%)	28(68.3%)	91(65.9%)
	LBM	31(32.0%)	12(29.3%)	43(31.2%)
	OP	3(3.1%)	1(2.4%)	4(2.9%)
χ^2^		33.564	23.250	56.618
*P* value		0.000*	0.000*	0.000*

*Notes*: All *P < 0.05

*Abbreviations*: *NBG* Normal blood glucose group; *ABG* Abnormal blood glucose group; *DXA* Dual energy X-ray absorptiometry; *QCT* Quantitative computed tomography; *BMD* Bone mineral density; *NBM* Normal bone mass; *LBM* Low bone mass; *OP* Osteoporosis

### Factors associated with BMD in middle-aged and elderly men

The BMD of L2-4 and FN detected by DXA and BMD of vertebrae detected by QCT, as dependent variables respectively, were engaged in the analysis of relationship between BMD and various factors, as a result of which different factors were adjusted and different models were established (Table 4). Independent variables, BMI, Cr, ALT, ABG and HbA1c, were involved in the regression model 1, in which BMD of FN by DXA is positively correlated with BMI (β = 0.230, *P* < 0.05) and BMD by QCT is negatively correlated with ABG (β = -0.217, *P* < 0.05). Besides, other indicators in model 1 did not reach statistical differences. Moreover, age, as an independent variable, was added to the regression model 2 on the basis of model 1. The results showed that age is positively correlated with BMD of L2-4 by DXA (β = -0.217, *P* < 0.05).

**Table 4:** Multiple linear regression analysis of influencing factors associated with BMD (β value)

	DXA BMD				QCT BMD	
	L2-4		FN			
	Model 1	Model 2	Model 1	Model 2	Model 1	Model 2
BMI	0.093	0.066	0.230*	0.161	0.109	-0.019
Cr	0.215	0.172	0.035	0.103	0.070	0.197
ALT	0.048	0.169	0.060	0.009	0.033	-0.063
ABG	-0.029	-0.071	-0.001	0.050	-0.217*	-0.119
HbA1c	0.198	0.134	-0.015	0.030	-0.019	0.065
Age	0.093	0.066	0.230*	0.161	0.109	-0.019

*Notes*: Model 1: BMI, Cr, ALT, ABG and HbA1c were plug into the regression equation; Model 2: all variables in model1 + Age. The β values in the table are all with *P < 0.05

*Abbreviations*: *DXA* Dual energy X-ray absorptiometry; *QCT* Quantitative computed tomography; *BMD* Bone mineral density; *L2-4* Lumbar 2–4; *FN* Neck of femur; *BMI* Body mass index; *Cr* Creatinine; *ALT* Glutamic-pyruvic transaminase; *ABG* abnormal blood glucose; *HbA1c* Glycated hemoglobin

## Discussion

Results of this study suggested that BMD measured by QCT was lower in middle-aged and elderly men with ABG than that in NBG patients, which was not consistent with the BMD measured by DXA. The detection rates of OP and LBM in middle-aged and elderly men by using QCT method were significantly higher than that by DXA. Whether in the ABG group or the NBG group, the QCT method has a significant advantage over DXA.^8^ However, there is no significant difference of the detection rates between ABG and NBG groups by both the QCT and DXA methods. A negative effect of ABG on BMD measured by QCT was found, while that did not reach statistical difference after correction of age.

Osteoporosis is a disease from a complex etiology including genetic and environmental factors.^9,10^ The prevalence of OP increases with increasing age, while the correlation between age and BMD were inconsistent in previous researches, especially in the lumbar spine BMD of elderly men.^11^ Although it is generally identified that the BMD of lumbar spine increases with age,^12,13^ there were some studies revealed that BMD of the lumbar spine in men gradually decreases with age^14^ and stabilizes or slightly increases until the age of 75–89. These results are almost based on BMD measured by DXA. The homologous contradictory findings by DXA measurement were also shown in our study, in which lumbar BMD was positively correlated with age (β = 0.281) while FN BMD was negatively correlated with age (β = -0.297). The discordance may be caused by the reasons that the areal density measured by DXA which might be affected by the size and shape of the bone. The sites of lumbar BMD measured by DXA consist of vertebral bodies and its rear appendages. As the trabecular bone and cortical bone with distinct bone turnover rates cannot be measured by DXA separately, the values detected are susceptible to lumbar degenerative disease and abdominal aortic calcification and consequently leading to pseudo increase of BMD.^15^ Thus, FN BMD, compared with lumbar spine BMD, detected by DXA could better be the representation of changes in male BMD with increasing age.^16^ Nevertheless, volumetric trabecular bone density measured by QCT is not superimposed by cortical bone and other tissues, revealing the actual lumbar BMD. Our results indicated that BMD detected by QCT is negatively related with age, trend of which was similar with FN BMD. While the higher β value (-0.558) of QCT measurement suggested that more decrease of BMD could be detected by QCT than FN DXA with increase of age under control of other factors. At the same time, higher detection rates of LBM and OP by QCT reflect a higher sensitivity of BMD detection in middle-aged and elderly men.

T2DM or pre-diabetes state, including impaired glucose tolerance and impaired fasting glucose, has a negative impact on bone metabolism. Patients with T2DM has an increased risk of vertebral and hip fractures by 1.7–2.2 times compared with those without DM, and even 2.0–3.4 times increased risk occur in the patients with more than 15 years diabetes course.^14^ However, some existing studies associated with DXA measurement suggested that increase of BMD in T2DM patients showed no significant difference with non-diabetes patients^17^, which was in accordance with the present results that BMD by DXA of either lumbar spine or femoral neck in ABG group did not significantly differ from NBG group. BMD in T2DM patients detected by DXA does not seem to forecast the increased risk of fracture. While studies associated with QCT measurement demonstrated that increase of BMD in T2DM was mainly concentrated in trabecular bone certified by QCT, revealing decreased BMD in T2DM patients could be manifested by QCT.^16,18,19^ In accordance with previous study, our results illustrated that the BMD measured by QCT was significantly lower in the ABG group than in the NBG group (*P* < 0.05), and negative correlation between ABG and QCT BMD was proved by multiple linear regression analysis (β = -0.127, *P* < 0.05). The hereinbefore further reveals that QCT is better than DXA in detecting BMD of middle-aged and elderly men with abnormal glucose metabolism. However, the correlation between ABG and QCT BMD did not reach a statistical difference after adjusting for age, hinting that age may contribute more to the change of BMD than ABG. Our study conducting comparison of BMD detected by QCT and DXA in NBG and ABG patients revealed that detection rates of LBM and OP by QCT were higher than by DXA, while no significant difference was observed between two groups. These results indicated that QCT showed higher sensitivity in the diagnosis of osteoporosis than DXA in middle-aged and elderly men, but it did not show an advantage in ABG individuals. This may be due to some limitations of our study. First, more clinical trials are needed to validate the findings because of the small sample size of the study. Then, the cases of patients with abnormal glucose metabolism were less, which may lead to bias. In the follow-up study, the sample size needs to be expanded, and the inclusion of relevant indicators should be improved to further verify the conclusion.

## Conclusion

In conclusion, the detection rate by QCT in middle-aged and elderly men with different glucose metabolism is higher than that by DXA. The abnormal blood glucose metabolism is negatively correlated with BMD measured by QCT, which is affected by age. The conclusion needs to be verified in a larger sample study.

## Data Availability

The datasets used and/or analysed during the current study available from the corresponding author on reasonable request.

## Abbreviations

QCT: Quantitative computed tomography
BMD: Bone mineral density
OP: Osteoporosis
ALT: Alanine transaminase
sCa: Serum calcium
HbA1c: Glycated hemoglobin
25OHD: 25-Hydroxy vitamin D
CTX: Cross linked C telopeptide of type 1 collagen
2hPG: 2-Hour postprandial plasma glucose
DXA: Dual energy X-ray absorptiometry
T2DM: Type 2 diabetes mellitus
FPG: Fasting plasma glucose
Cr: Creatinine
sPi: Serum inorganic phosphorus
PTH: Parathyroid hormone
FN: Femur neck
P1NP: Amino-terminal propeptide of type 1 collagen
ISCD: International Society for Clinical Densitometry
